# Sensorimotor adaptation of voice fundamental frequency in Parkinson's disease

**DOI:** 10.1371/journal.pone.0191839

**Published:** 2018-01-26

**Authors:** Defne Abur, Rosemary A. Lester-Smith, Ayoub Daliri, Ashling A. Lupiani, Frank H. Guenther, Cara E. Stepp

**Affiliations:** 1 Department of Speech, Language, and Hearing Sciences, Boston University, Boston, MA, United States of America; 2 Department of Biomedical Engineering, Boston University, Boston, MA, United States of America; 3 Department of Otolaryngology–Head and Neck Surgery, Boston University School of Medicine, Boston, MA, United States of America; Northwestern University, UNITED STATES

## Abstract

**Objective:**

This study examined adaptive responses to auditory perturbation of fundamental frequency (*f*_o_) in speakers with Parkinson’s disease (PD) and control speakers.

**Method:**

Sixteen speakers with PD and nineteen control speakers produced sustained vowels while they received perturbed auditory feedback (i.e., *f*_o_ shifted upward or downward). Speakers’ pitch acuity was quantified using a just-noticeable-difference (JND) paradigm. Twelve listeners provided estimates of the speech intelligibility for speakers with PD.

**Results:**

Fifteen responses from each speaker group for each shift direction were included in analyses. While control speakers generally showed consistent adaptive responses opposing the perturbation, speakers with PD showed no compensation on average, with individual PD speakers showing highly variable responses. In the PD group, the degree of compensation was not significantly correlated with age, disease progression, pitch acuity, or intelligibility.

**Conclusions:**

These findings indicate reduced adaptation to sustained *f*_o_ perturbation and higher variability in PD compared to control participants. No significant differences were seen in pitch acuity between groups, suggesting that the *f*_o_ adaptation deficit in PD is not the result of purely perceptual mechanisms.

**Significance:**

These results suggest there is an impairment in vocal motor control in PD. Building on these results, contributions can be made to developing targeted voice treatments for PD.

## Introduction

Parkinson’s disease (PD) is a slowly progressing neurodegenerative condition. It affects approximately 3–5% of the population over the age of 85 years [1], with a prevalence that is predicted to increase drastically [2]. In addition to the cardinal motor deficits such as bradykinesia, rigidity, tremor, and postural instability, over 90% of individuals with PD develop hypokinetic dysarthria, a motor speech disorder [3]. Hypokinetic dysarthria is primarily distinguished by reduced speech loudness [4], but also frequently encompasses dysprosody, which can involve ‘monopitch’, or the perception of reduced fluctuation in fundamental frequency (*f*_o_) during speech production [5, 6]. These disruptions in typical prosodic patterns in speakers with PD have been shown to negatively influence listeners’ perceptions of the speakers’ tone, personality, and mood [7] and to correlate to listeners’ perception of the ‘naturalness’ of their speech [8]. Given the importance of prosody for speech intelligibility and social communication in PD [9–12], it is necessary to better characterize the underlying causes of prosodic impairments in order to improve the assessment and treatment of speech in PD. This study aims to investigate the patterns of sensorimotor adaptation to perturbation of voice *f*_o_ in speakers with PD to clarify deficits in *f*_o_ control in PD.

Many studies of auditory-motor control involve distortion of auditory feedback during speech. These studies have revealed that typical individuals generally show compensatory (opposing the direction of the perturbation) responses to both sustained and brief, unanticipated modifications in auditory feedback. Studies show that as feedback of voice *f*_o_ is increased gradually over many productions (i.e., a sustained perturbation), speakers show compensatory adaptive responses by gradually lowering their *f*_o_ across the experiment [13–18]. Studies involving brief, unanticipated perturbation of auditory feedback similarly showed that speakers had compensatory reflexive responses: as *f*_o_ is suddenly increased, speakers respond by quickly lowering their *f*_o_ [19, 20]. The adaptive responses to sustained modifications and the reflexive responses to sudden modifications observed in these experiments can be accounted for by the Directions Into Velocities of Articulators (DIVA) model, a neural network model of speech motor skill acquisition and speech production.

The DIVA model [21, 22] is a widely used characterization of the neural and computational processes underlying speech production that describes how feedforward and feedback control processes are combined to produce speech movements. Similar to other modern theories of speech motor control [23, 24], DIVA posits there are separate processes governing perception of auditory feedback and the creation of future vocal motor actions. According to the DIVA model, an unexpected perturbation of auditory feedback during speech leads to a mismatch between expected and perceived auditory signals; if this “auditory error signal” passes a certain threshold, it is then transformed into a corrective motor command by the auditory feedback control system, resulting in a reflexive response as described above [25, 26]. Although similar to DIVA, the State Feedback Controller (SFC) model for speech [23] states that when auditory feedback is received, there is also an internal model taking into account vocal tract controls. Furthermore, in the SFC model, errors are continuously generated (not solely when the auditory error signal has reached a specific threshold, as in DIVA). Regardless, if a perturbation is maintained for a sustained period of time, corrective commands generated by the auditory feedback control system become subsumed into feedforward commands; in other words, the feedforward controller *adapts* to the sustained perturbation [e.g.,^15^, ^16^, ^27^–^29^].

In the speech domain, reflexive responses to sudden, brief, perturbations of the acoustical characteristics of vowels [i.e. formants; 30] in PD have revealed significantly reduced compensatory gain (the magnitude of the compensation divided by the magnitude of the feedback shift) compared to controls during formant changes of 15% but comparable compensatory gain between groups for larger formant changes of 30%. One study thus far investigated adaptive responses to sustained alterations in formants in speakers with PD [31]. Speakers with PD had reduced adaptive responses to a 30% perturbation compared to healthy control speakers when analyzed on a group level. Thus, during reflexive formant changes of 30%, PD speakers show comparable responses relative to controls, whereas adaptive responses to gradual formant changes of 30% show reduced responses in PD speakers relative to controls. Reflexive responses to sudden, brief, perturbations of *f*_o_ in PD have revealed larger reflexive response magnitudes [32, 33] and compensatory gains [30] to perturbations and longer response durations [34] relative to control speakers.

Articulatory motor control (which primarily affects formants) may differ from control of vocal parameters such as *f*_o_ [21, 30, 35, 36]. Furthermore, vocal characteristics in PD appear to have a different timeline compared with articulatory symptoms [37–39]. Therefore, it is necessary to characterize adaptive responses to sustained perturbations to both articulatory perturbations and vocal parameters such as *f*_o_ in PD.

To our knowledge, adaptive responses to sustained perturbations of *f*_o_ have not yet been studied in PD. In the current work, auditory-motor control of *f*_o_ was investigated by comparing adaptive responses to a sustained 100 cent perturbation of *f*_o_ (implemented gradually over time) in speakers with PD relative to control speakers. A cent is a logarithmic unit of measure of changes in frequency, like the semitone; 100 cents are equivalent to 1 semitone. We hypothesized that speakers with PD would show reduced adaptation to sustained perturbation of *f*_o_ as compared to control speakers due to disordered sensorimotor integration. Furthermore, based on previous findings that individuals with better pitch acuity had larger adaptive responses to formant perturbation [29], we hypothesized that, in the current study, individuals with better pitch acuity would have a larger degree of compensation to perturbations in *f*_o_. We also hypothesized that speakers with PD who had less intelligible speech, suggesting impaired vocal prosody, would have reduced adaptive responses due to a smaller vocal pitch range. Lastly, given the evidence for variable onset times of voice difficulties in the progression of PD, we did not expect disease severity to be a predictor of adaptive responses.

## Materials and methods

### Participants

All participants provided written consent in compliance with the Boston University Institutional Review Board under protocol 2625, which approved this study. Twenty-one speakers with PD and twenty-eight control speakers were recruited to participate in the study. Four speakers with PD were excluded from the study group due to abnormal hearing thresholds for older adults (N = 2, see Hearing Thresholds) or inability to complete the main experimental task (N = 2). Eight control speakers were excluded from the study group due abnormal hearing thresholds (N = 6), perceptually atypical voice (N = 1), or failure of a pre-study cognitive screening (N = 1). One speaker with PD and one control speaker were excluded due to data analyses restrictions on the primary experimental task (see Data Analysis). Thus, sixteen speakers with PD aged 52–73 years (7 female) and nineteen healthy control speakers aged 50–77 years (8 female) were included in the study (see Table 1). The complete study lasted two sessions. Fourteen speakers with PD and eleven control speakers completed both sessions, and two speakers with PD and eight control speakers completed a single session. All speakers with PD were diagnosed with idiopathic PD by a neurologist prior to participation in the study and were receiving daily levodopa/carbidopa therapy. Twelve naïve listeners aged 18–23 years (6 female) were included in the study to assess the speech intelligibility of the speakers with PD (see Data Analysis). Control speakers and listeners denied any known neurological, speech, hearing, cognitive, or language disorders.

**Table 1:** Participant characteristics (M: mean, SD: standard deviation).

	Speakers with PD	Control Speakers	Listeners
**Age (years)**	M = 64.8, SD = 6.3 Range: 52–73	M = 65.3, SD = 4.6 Range: 50–77	M = 21.9, SD = 2.9 Range: 18–23
**Sex**	8 F, 8 M	10 F, 9 M	6 F, 6 M
**Time since diagnosis (years)**	M = 6.97, SD = 7.05 Range: 1.5–30	N/A	N/A
**Hoehn and Yahr**	M = 2.1, SD = 0.6 Range: 1–3	N/A	N/A
**UPDRS Part III score (total motor score)**	M = 41.0, SD = 13.6 Range: 22–72	N/A	N/A

### Movement Disorder Society Unified Parkinson’s Disease Rating Scale (MDS-UPDRS)

Part III (Motor Examination) of the Movement Disorder Society sponsored revision of the Unified Parkinson’s Disease Ratings Scale [MDS-UPDRS; 40] was provided to each participant with PD to assess the motor signs of PD. This documents the severity of abnormalities during execution of motor function tasks such as speech, upper and lower extremity movements, and walking. The first author, certified to administer MDS-UPDRS, scored each examination per protocol. The UPDRS total motor score (see Table 1) was used as a correlate of disease progression.

### Hearing thresholds

Each participant underwent pure-tone hearing threshold testing at 250, 500, 1000, 1500, 2000, 3000, and 4000 Hz using 3M^TM^ E-A-RTONE^TM^ Gold 3A insert earphones and the Grason-Stadler GSI 18 Screening Audiometer. All participants had hearing thresholds within normal range for older adults [under 25 dB HL for frequencies 1000 Hz and below, and under 40 dB HL above 1000 Hz; 41]. None of the participants wore hearing aids.

### *f*_o_ Adaptation procedure

All experimental sessions were conducted in a sound-attenuating audiometric booth. Participants were seated in front of a computer monitor and wore a Shure omnidirectional MX153 earset microphone positioned at approximately 45 degrees from the midline and 7 cm from the corner of the mouth. The microphone signal was amplified via an RME Quadmic II microphone preamplifier and digitized via a MOTU Ultralite-mk3 Hybrid soundcard with a sampling rate of 48000 Hz. The auditory feedback was amplified at least 5 dB relative to the sound pressure level (SPL) at the microphone [based on relative dB between microphone and ear; 42]. Due to the length of the experimental procedure (3 conditions × 30 minutes each), sound exposure was kept to the minimum possible sound pressure level that allowed participants to adapt to auditory feedback changes. For calibration, the intensity of the auditory feedback in the headphones was measured using an IEC 60318–1 coupler (Type 4153, Bruel & Kjaer Inc., Norcross, GA) connected to a sound level meter (Type 2250 Hand Held Analyzer with Type 4947 ½” Pressure Field Microphone, Bruel & Kjaer Inc., Norcross, GA) such that an electrolarynx (TruTone Artificial Larynx, Griffin Laboratories, Temecula, CA) input with an intensity of 75 dB SPL approximately 7 cm from the microphone resulted in at least 80 dB SPL output in the headphones. Auditory feedback was administered via Sennheiser HD 280 Pro headphones, which attenuate air-conducted sound by approximately 32 dB (Sennheiser Electronic Corporation). The Sennheiser HD 280 Pro headphone set used across participants had a different response for some PD and control participants, but data were analyzed for adaptation response differences and there was no clear effect of the headphone response. The *f*_o_ was manipulated using Audapter [43], a MATLAB software package for configurable real-time manipulation of acoustic parameters of speech, using the ASIO4ALL driver. The total processing delay was 45 ms, which is below the threshold at which delayed auditory feedback can cause speech dysfluency [44].

The adaptive responses to *f*_o_ perturbation were recorded under three conditions: ‘‘shift-up”, “shift-down”, and “control”, with the order counterbalanced across participants. Each condition consisted of 160 trials and each trial was 11 s in duration. Participants were instructed to produce a sustained /α/ for 3 s when “aaa” was displayed in green on the computer monitor and to then rest their voice for the remaining 8 s until the next trial began. The “shift-up” and “shift-down” conditions consisted of trials over four ordered stages: baseline, ramp, hold, and after-effect. In the “shift-up” condition, the first 20 utterances, referred to as the *baseline*, were produced while receiving typical (unperturbed) feedback. In the following 60 trials, referred to as the *ramp*, the *f*_o_ in the auditory feedback increased by 1.69 cents with each successive trial, reaching a total level of 100 cents of perturbation above the participant’s true *f*_o_. For the next 40 trials, referred to as the *hold*, the *f*_o_ of the auditory feedback was maintained at the level of +100 cents of perturbation. In the last 40 trials, referred to as the *after-effect*, the auditory feedback was the same as the baseline (i.e., there was no *f*_o_ perturbation). The “shift-down” condition was identical, except that, during the ramp, the *f*_o_ of the auditory feedback decreased by 1.6 cents in each successive utterance, reaching a total level of -100 cents of perturbation by the end of the ramp. Perturbations during the ramp and hold phases were applied throughout the entire period of voicing. In the “control” condition, participants received their typical (unperturbed) feedback during all 160 trials (similar to the baseline and after-effect periods of the “shift-up” and “shift-down” conditions). The participants did not receive any information about differences among the three conditions.

### Pitch acuity

All participants completed a pitch discrimination task to assess auditory acuity to *f*_o_, or pitch acuity. Each trial consisted of two pure tones, presented via Sennheiser HD 280 Pro headphones. Each tone was 2 seconds in duration. The tones in each trial were separated by approximately 20 ms and were presented in random order. Stimulus presentation was controlled using a custom-written MATLAB (The Mathworks, Inc., 2013, Version 8.1.0.604 [R2013b]) script. Tones were played at an intensity of 65 dB, calibrated using the same method as the adaptation procedure but using the MATLAB generated stimuli as the input. Participants were asked to judge whether the tones were the ‘same’ or ‘different’. The initial difference between the two tones was 40 cents (10 Hz) with a reference tone of 440 Hz. Two consecutive correct judgments resulted in a progressively smaller difference (downstep) between the two tones in subsequent trials, whereas an incorrect judgment resulted in a progressively larger difference (upstep) between subsequent sets of tones, with a set ratio of downstep = upstep/2.448. The magnitude of the upstep was initially 20 cents (5 Hz). As trial number increased, the upstep decreased to 10 cents (2.5 Hz), 5 cents (1.27 Hz), and 1 cent (0.25 Hz), at the tenth, twentieth, and thirtieth trial, respectively. The experiment continued until there were 16 ‘reversals’ (an upstep followed by a downstep, or vice versa). Catch trials with no difference between the two tones were presented pseudo-randomly in 33% of trials to ensure data validity and attention to the task. Each participant performed between 36 and 85 trials, with an average of 70 trials. The duration of this task was 6–10 min.

### Speech Intelligibility Test (SIT)

Speakers with PD completed a randomly generated short Speech Intelligibility Test [SIT; 45] consisting of a set of 11 sentences increasing in word count from 5 to 15 words per sentence. Speakers were instructed to read the sentences in their typical speaking voice. Using the same recording equipment as the adaptation paradigm, speech was recorded in Audacity (Audacity Team, 2012, Audacity®. Version 2.0.0. audio editor and recorder).

### Data analysis

#### Adaptive responses

To determine adaptive responses, the mean *f*_o_ was calculated over each trial using an autocorrelation method via Praat (Boersma & Weenink, 2014, Version 5.4.08 –Version 6.0) scripts. All trials were inspected and manually corrected for tracking issues or noise by selecting a stable portion of the signal with a minimum duration of 1 second. The mean *f*_o_ of each selected portion was converted to cents, using the mean *f*_o_ of the baseline of that condition as the reference frequency. In order to account for normal variation in *f*_o_ over the course of 160 trials, the *f*_o_ during the control condition in which no perturbation was applied was subtracted from the “shift-up” and “shift-down” conditions to determine the resulting adaptive responses. Data from one speaker with PD and one control speaker were excluded due to unstable vocal control during the control condition. Unstable vocal control was defined as a greater than 300 cents range within one phase (baseline, ramp, hold, and/or after-effect). Since the control condition was used to normalize the “shift-up” and “shift-down” conditions, a 300 cents or greater variability in the control condition was determined to result in adaptive responses that were likely not representative. Two additional control speakers had unstable vocalizations during the control condition; however, they were able to return to repeat the task another day. Likewise, three control speakers experienced equipment failures during one of their conditions and repeated those conditions. In total, eleven control speakers and fourteen speakers with PD completed all three conditions: “shift-up”, “shift-down”, and control. Eight control speakers and two speakers with PD were only able to complete the control condition and either the “shift-up” or “shift-down” condition. Given the counterbalancing of condition order and excluded participants, fifteen total adaptive responses were successfully recorded and used in analyses for each “shift-up” and “shift-down” condition for both the control and PD group. The two-way mixed-model analyses of variance (ANOVAs) were performed on the “shift-up” and “shift-down” adaptive responses to assess the effects of group (between-participant; speakers with PD vs. control speakers), phase (within-participant; baseline, ramp, hold, after-effect), and their interaction. Factor effect sizes were quantified using the squared partial curvilinear correlation, η_p_^2^. An alpha of 0.05 or less was determined to be statistically significant. In order to ensure that group differences in the adaptive responses were not driven by potential differences in the control condition that was used in the response calculations, the *f*_o_ variability between groups during the control (no perturbation) condition was assessed using a two-sample, two sided, *t*-test comparing the average variance over the course of the 160 control trials for each participant by group.

Two-tailed Student’s *t*-tests were used to determine whether each individual adaptive response during the hold phase was significantly different than zero (no response). These were performed for the adaptive responses (normalized to the control condition) of both the “shift-up” and “shift-down” conditions. An alpha of 0.05 or less was determined to be statistically significant. Given the nature of this individual investigation, corrections for alpha inflation were not applied to account for the multiple tests. After group analysis, individual responses were classified as following, compensating, or non-responsive. For the “shift-up” condition, individual adaptive responses were termed as compensating if they were statistically less than zero and as following if they were statistically greater than zero. For the “shift-down” condition, individual adaptive responses were termed as compensating if they were statistically greater than zero and as following if they were statistically less than zero. In both conditions, responses were termed as unresponsive if they were not statistically different from zero.

The average degree of compensation during the hold phase of the “shift-up” and “shift-down” adaptive tasks was calculated as the mean of the response during the hold phase for “shift-up” responses and the additive inverse of the mean of the response during the hold phase for “shift-down” responses (so that larger positive values can be interpreted as larger degrees of compensation, regardless of condition). In the PD group, the average degree of compensation for both shift directions was compared to participant age, years since diagnosis, UPDRS total motor score, intelligibility, and pitch acuity using Pearson-product moment correlation coefficients.

Previous studies have found a relationship between *f*_o_ variability in the baseline (unperturbed) phase and the amount of compensation during brief, unexpected perturbations to *f*_o_ in both control speakers [46] and speakers with PD [47]. Thus, to investigate the effects of vocal *f*_o_ control in the baseline phase in the current work, the average degree of compensation was compared to baseline *f*_o_ variability for both the “shift-up” and “shift-down” conditions for both participant groups using Pearson-product moment correlation coefficients. The baseline *f*_o_ variability was calculated by determining the *f*_o_ variability across a 200 ms period of each trial of the baseline phase in cents, using the mean *f*_o_ of that trial as a reference frequency, and taking the average *f*_o_ variability of all 20 baseline trials. In both the PD and control group, the average degree of compensation for both shift directions was compared to their average baseline *f*_o_ variability using Pearson-product moment correlation coefficients.

#### Pitch acuity

Pitch acuity of each speaker was quantified as a just-noticeable-difference (JND) in cents and was calculated by averaging data from the last six reversals in the adaptive forced-choice procedure. This estimated the degree of *f*_o_ difference that could be detected by the participant with 70.9% accuracy [48]. A two-sample, two-sided, Student’s *t*-test was used to compare pitch JND values between all 19 control speakers and all 16 speakers with PD.

A Bonferroni correction was applied to the interpretation of the *t*-tests and Pearson’s correlation *p*-values to account for the 16 multiple comparisons (1 *t*-test for *f*_o_ variability during the control condition, 1 *t*-test for pitch JND values, and 14 correlations) applied at the group level, such that an alpha of 0.05/16 = 0.00313 or less was determined to be statistically significant.

#### Speech Intelligibility Test (SIT)

Of the 16 speakers with PD, one speaker did not complete the SIT due to having missed the second session. The remaining 15 SIT speech recordings from the PD group were normalized to have equal amplitudes and were then combined with multi-speaker babble from five male and five female voices using a custom script in MATLAB (Mathworks, 2013 Version 8.1.0.604 [R2013a]). The resulting signal-to-noise ratio (SNR) was 3.5 dB. Stimuli were presented via Sennheiser HD 280 Pro headphones at a level of 70 dB SPL. The calibration procedure was the same as described for the adaptation procedure but the normalized speech samples playing from the MATLAB script were used as the input. Twelve listeners were instructed to listen to recordings and transcribe what they heard to the best of their ability after listening to each speech sample a maximum of two times. The order of presentation of the speech samples was randomized per listener to prevent order bias. Initial intelligibility scores for the listener transcriptions of the SIT sentences were calculated with a custom script in MATLAB (Mathworks, 2013 Version 8.1.0.604 [R2013a]). Scores were calculated as the total words matching phonemically between the listener transcription and the SIT sentences divided by the total number of words [49, 50]. A document was generated with calculated scores and was then hand-checked for each listener to include misspellings and homonyms as correct [49]. The intelligibility score used in further analyses was the SIT sentence scores of each speaker with PD averaged over all listeners and sentences.

## Results

**Fig 1:**
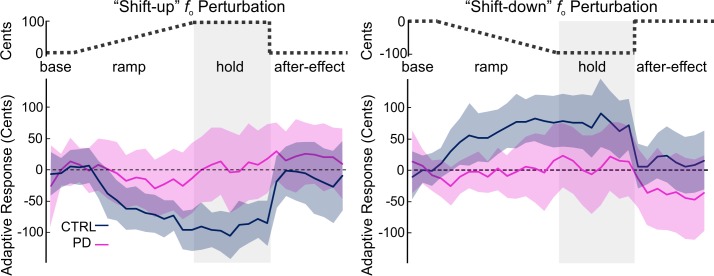
Average adaptive responses by group. An adaptive “shift-up” (left) and “shift-down” (right) perturbation was applied to the *f*_o_ of auditory feedback with a maximum perturbation of 100 cents or -100 cents respectively (schematized in upper panel). Mean adaptive responses in cents are plotted as the mean across five-trial blocks for control speakers (dark blue line) and speakers with Parkinson’s disease (light pink line) with shading indicating 95% confidence intervals.

Consistent with previous studies in typical speakers [13, 15], control speakers generally compensated in the sensorimotor adaptation task. Control speakers generally decreased their *f*_o_ during the ramp and hold phases of their “shift-up” responses and increased their *f*_o_ during the ramp and hold phases of their “shift-down” responses (see Fig 1). At a group level, our results in speakers with PD are consistent with previous work showing reduced compensations in speakers with PD relative to control speakers during sustained vowel formant perturbation [31]; the speakers with PD had significantly reduced compensatory responses to *f*_o_ as compared to control speakers in both perturbation shift directions (Fig 1). Overall, the adaptive responses showed significant interactions between group and phase for both “shift-up” (η^2^_p_ **=** 0.06, F = 19.9, p < 0.001) and “shift-down” paradigms (η^2^_p_ = 0.04, F = 12.4, p < 0.001). These effects were not driven by differences in the control condition: there was not a statistically significant difference in *f*_o_ variability between the two groups during the control condition, (*p* = 0.36).

**Fig 2:**
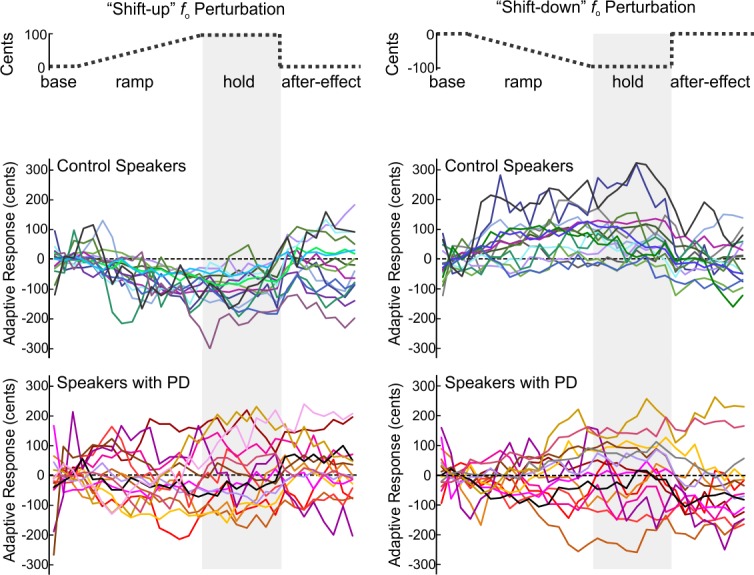
Individual adaptive responses by group. Individual adaptive responses in cents for “shift-up” (left) and “shift-down” (right) perturbations (schematized in upper panel). Responses are plotted as the mean across five-trial blocks for fifteen adaptive responses from control speakers (upper) and fifteen adaptive responses from speakers with Parkinson’s disease (lower).

During the hold phase, although the control speakers showed average compensatory responses of -91 cents (SD = 59 cents) for “shift-up” and 80 cents (SD = 84 cents) for “shift-down”, the average responses of speakers with PD were only 7 cents (SD = 107 cents) for “shift-up” and 8 cents (SD = 110 cents) for “shift-down”. However, at an individual level, the responses are qualitatively different between control speakers and speakers with PD: while most control speakers showed compensation to perturbations in *f*_o_, speakers with PD displayed highly variable responses spanning both the compensatory and following (i.e., the same direction as the perturbation) directions (see Fig 2). For the “shift-up” condition, the PD group had seven compensatory, three non-responsive, and five following responses. In the control group, fourteen of the fifteen “shift-up” responses were compensatory; one was non-responsive and no responses were following. For the “shift-down” condition, the PD group had seven compensatory, three non-responsive, and five following responses. In the control group, twelve responses were compensatory, two were non-responsive, and one response was following.

**Fig 3:**
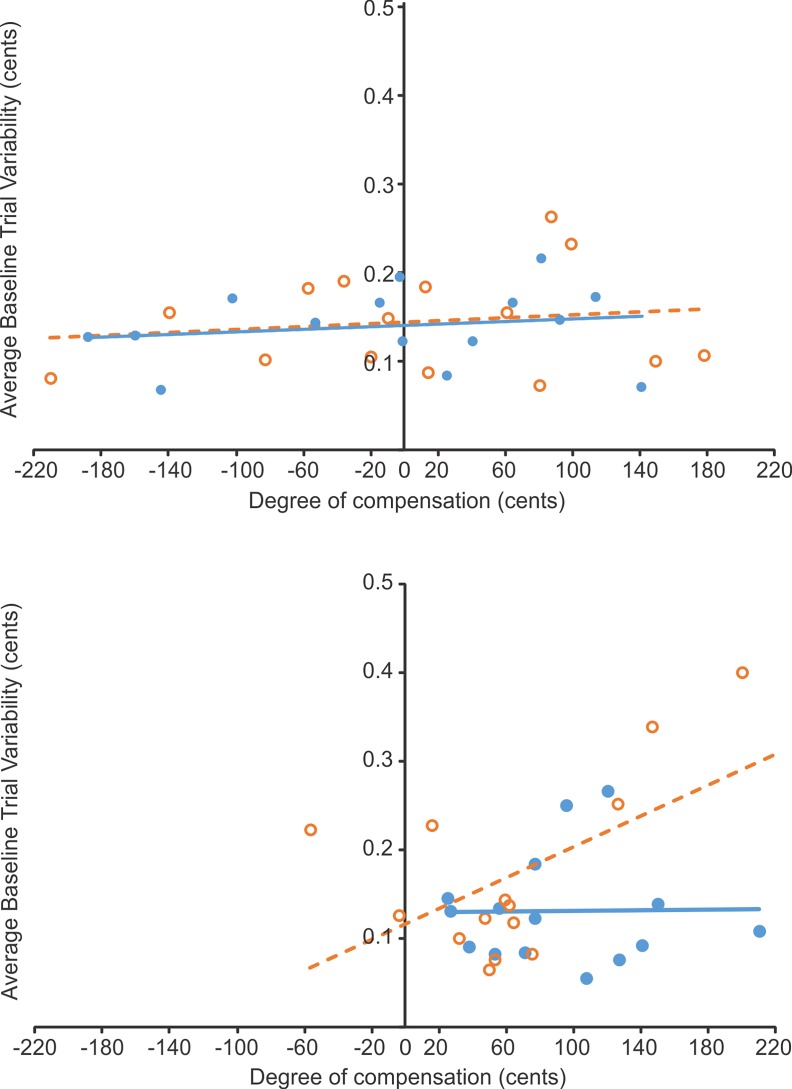
Baseline *f*_o_ variability compared to the degree of compensation. Relationship between baseline *f*_o_ variability (cents) and degree of compensation (cents) for speakers with PD (upper panel) and for control speakers (lower panel) during the “shift-up” (blue, closed circles) and “shift-down” (orange, open circles) conditions. Lines of best fit are plotted for the “shift-up” (blue solid line) and the “shift-down” (orange solid line) conditions.

The Pearson-product moment correlation between baseline *f*_o_ variability and degree of compensation, for both the “shift-up” and “shift-down” conditions are listed in Table 2. For both the “shift-up” and “shift-down” conditions, no significant correlations were found between baseline *f*_o_ variability and degree of compensation in either speaker group (Fig 3).

**Table 2:** Pearson’s product-moment correlations between the degree of compensation and baseline *f*_o_ variability.

	“shift-up” compensation	“shift-down” compensation
Control group baseline *f*_o_ variability	0.13 (*p* = 0.64)	0.57 (*p* = 0.03)
PD group baseline *f*_o_ variability	0.34 (*p* = 0.22)	-0.08 (*p* = 0.78)

The average pitch JND of control speakers was 48 cents (SD = 52 cents) and the average for speakers with PD was 52 cents (SD = 51 cents); there was not a statistically significant difference between the two groups (*df* = 32, T = -0.19, *p* = 0.85).

The heterogeneity in the adaptive responses of speakers in the PD group was not explained by any of the catalogued descriptors. The Pearson’s correlations between the degree of compensation during the hold phase of the “shift-up” and “shift-down” tasks and age, years since diagnosis, UPDRS total motor score, pitch JND, and intelligibility were all nonsignificant (*p* > 0.0033; see Table 3). Given our conservative correction for multiple comparisons, it is worth noting that, although non-significant, there were a few substantial trends. The average degree of compensation during “shift-up” was associated with intelligibility (*r* = -0.45, *p* = 0.10), with less intelligible individuals compensating to a greater degree. The average degree of compensation during “shift-down” was associated with age (*r* = -0.53, *p* = 0.04); younger participants tended to have a greater degree of compensation than older participants.

**Table 3:** Pearson’s product-moment correlations for speakers with PD.

	“shift-up” compensation	“shift-down” compensation
age	0.31 (*p* = 0.27)	-0.53 (*p* = 0.04)
years post-diagnosis	0.27 (*p* = 0.33)	-0.02 (*p* = 0.93)
UPDRS total motor score	0.24 (*p* = 0.38)	0.03 (*p* = 0.33)
pitch JND	0.25 (*p* = 0.36)	-0.34 (*p* = 0.21)
intelligibility	-0.45 (*p* = 0.10)	0.08 (*p* = 0.78)

## Discussion

### Adaptive responses to *f*_o_ perturbation in PD

Sensorimotor adaptation has been studied extensively in PD, but only one prior study investigated sensorimotor adaptation in PD in the speech domain [31]. Adaptive responses to perturbations of the first formant of vowels (F1) during word productions were analyzed and reported at a group level. The study found reduced responses in the PD group compared to the healthy control group [31]. The data presented here, at a group level, extend this finding to the control of *f*_o_. Furthermore, on an individual level, these data demonstrate qualitatively different responses between control speakers and speakers with PD. In speakers with PD, responses were also not consistent among shift directions (ie, speakers who compensated in the shift down condition did not always compensate in the shift up condition or vice versa). While control speakers showed relatively consistent compensatory responses to perturbation, speakers with PD had highly variable responses, with either compensatory responses, no response, or even “following” responses (changing *f*_o_ in the same direction as the perturbation). There was no clear pattern in these responses. In the PD group, 2/15 subjects only completed one shift condition. Of the remaining 13/15 who completed both conditions, 7/13 had the same result regardless of direction (3 compensated, 2 did not respond, and 2 followed). The remaining 6/13 subjects who completed shifts in both directions did not show the same response in the two directions: 2 compensated in one direction and did not respond in the other, 3 compensated in one direction and followed in the other, and 1 followed in one direction and did not respond in the other. Using a reflexive paradigm, Kiran and Larson [34] also found more “following” reflexive responses to unanticipated perturbations of *f*_o_ in speakers with PD than in control speakers. However, the relationship between following reflexive and following adaptive response types in PD is unclear, especially given the lack of consensus between adaptive and reflexive response types in general.

While the group-level responses in the current findings align with those reported by Mollaei, Shiller [31], the individual responses differ. The heterogeneous results seen in the current work were an unexpected outcome of the study. These responses could be due to several differences between the current work and Mollaei, Shiller [31]: perturbing vocal vs. articulatory characteristics of speech (*f*_o_ vs. F1) or differences in how the perturbation was introduced and the experimental set up. Perturbations of *f*_o_ and F1 may involve different control systems. Based on short- and long-term effects of auditory deprivation, it has been hypothesized that so-called ‘postural’ parameters such as *f*_o_ are more heavily reliant on auditory feedback control processes than so-called ‘segmental’ parameters such as F1, which are more heavily reliant on feedforward control processes [51, 52]. This suggests that a potential feedforward system impairment in auditory-motor integration [refinement and updating of the feedforward system basedon on systematic changes in auditory feedback; e.g., 21] might have a greater effect on articulatory (F1) motor control relative to vocal (*f*_o_) motor control. Furthermore, reduced loudness is a hallmark feature in PD [4], which has a biomechanical relationship with *f*_o_ [53, 54]. Thus, some interaction between hypophonia and *f*_o_ in the speakers with PD could have contributed to the variability in *f*_o_ adaptive responses. Other than the parameter to which the perturbation was applied, there are additional methodological differences between the current study and Mollaei, Shiller [31]. The paradigm used by Mollaei, Shiller [31] differed from most current speech adaptation paradigms [15, 29, 55]: a ramp phase was not included to gradually introduce the perturbation. This paradigm difference is important to note when comparing the adaptive responses. Visuomotor studies have shown differences in responses to sudden (no ramp) and gradual introduction (with ramp) of perturbation in healthy controls [56, 57] and individuals with PD [58]. Lastly, the current study applied auditory perturbations in cents, which are a relative measure of frequency, rather than changing feedback by the same set frequency for all subjects. This is a common method used in *f*_o_ perturbation paradigms [16, 25, 30, 59] and ensures that all subjects perceive the same magnitude of feedback perturbation since the just-noticeable-difference for frequency acuity increases as a function of frequency [60, 61]. In formant perturbations, shifts are applied as a set frequency change in the vowel formants rather than a subject specific shift in cents [27, 62], which may contribute to variations in results between the current work and formant perturbations in PD [31].

The individual responses seen in the current study during the after-effect stage align well with a study of visuomotor control in PD. Contreras-Vidal and Buch [63] found heterogeneous responses in speakers with PD during the after-effect stage of a visuomotor adaptation task that were not seen in healthy controls. Similar heterogeneous responses were found in the current work during the after-effect stage in the PD group; however, these may have been a continuation of the heterogeneous responses that were also seen in the ramp and hold.

In terms of the relationship between baseline *f*_o_ variability and degree of compensation, the current results showed no significant correlations for either speaker group for both “shift-up” or “shift-down” conditions (Table 2). The results for speakers with PD are in contrast to Huang, Chen [47] who reported a significant positive correlation in a PD group between baseline *f*_o_ variability (during a 200 ms baseline phase) and the degree of compensation to unexpected *f*_o_ perturbations. The lack of significant correlations in the current work is likely due to the differences between so-called “reflexive” and “adaptive” responses to sensory perturbations. Such distinctions between unexpected *reflexive* shifts and studies of *adaptation* (repeated perturbation applied on many consecutive trials, causing changes in the sensorimotor control system that continue to affect movements for a number of trials after the perturbation has been removed) have been made in a large number of studies of both speech production [e.g. 15, 64–66] and motor control more generally [e.g. 56, 57, 58, 67–70].

### Interpretation of potential neurophysiological mechanisms

The atypical responses to gradual, repeated, *f*_o_ perturbation seen in the current study support the hypothesis that there may be an impairment in the auditory-motor learning process in PD. Using the DIVA and SFC models to interpret these findings, in neurotypical participants, the feedback system compares the output *f*_o_ to the target *f*_o_, and generates an error signal due to a mismatch between the two. In the case of the SFC model, an internal vocal tract model is also present and is continuously keeping track of the state of vocal articulators to use this information for future commands. If either the feedback controller or the internal model (in the case of SFC) register an error signal, a corrective motor command is generated to oppose the shift in *f*_o_. This is communicated to the feedforward subsystem which is adapted (over multiple perturbed productions) to reduce the mismatch between target and output in future productions. The heterogeneous responses seen in the adaptive responses of the individuals with PD suggest that either: 1) the *f*_o_ perturbation was not correctly detected by the auditory feedback controller (DIVA) or the internal vocal tract model (SFC), 2) detected auditory errors were not properly translated into corrective motor commands, or 3) the feedforward system did not correctly modify motor commands for subsequent productions by incorporating corrective motor commands from the auditory feedback controller. These possibilities are addressed in the following paragraphs.

If atypical adaptive responses were due to a problem with *f*_o_ detection in the auditory system in speakers with PD, this would suggest less sensitive pitch discrimination compared to healthy speakers. In the current results, there was no significant difference in pitch acuity between speakers with PD and control speakers. Additionally, pitch acuity was not correlated with the degree of compensation in adaptive responses in speakers with PD. Interpreting these results with the DIVA and SFC models, this indicates that there was not likely a deficit in detecting pitch changes in the speakers with PD. The pitch acuity findings in the current study are in contrast with previous work that found reduced ability to discriminate pitch in 12 individuals with PD compared to 15 healthy controls [71]. The conflicting results could potentially be explained by natural variation in musical experience of participants, paradigm differences, or the limited sample sizes employed by both studies. Previous work has shown that musicians may be better than non-musicians at discriminating pitch [72, 73]. Since neither Troche, Troche [71] or this current study reported musical training, it is possible that it contributed to differences between study results. Paradigm differences between Troche, Troche [71] and the current study could also explain the different findings. Among other differences, tones were presented with an inter-stimulus interval (ISI) of 750 ms in Troche, Troche [71]. In the current study, an ISI of only 20 ms was used. It is possible that the longer ISI employed by Troche, Troche [71] is responsible for the poorer pitch acuity seen in speakers with PD in that study: assessment procedures for frequency discrimination are known to be impacted by relative attentional and cognitive ability [74]. In fact, reduced cognitive function has specifically been shown to interact with ISI, resulting in differentially poorer frequency discrimination in individuals with Alzheimer’s disease when long ISIs are used [75]. Future studies comparing pitch acuity measures as a function of ISI in speakers with and without PD are necessary to confirm this. Finally, especially given the heterogeneity in PD presentation, differences between the two studies may simply be a function of sample size. That said, since pitch acuity was not correlated with the adaptive responses in the speakers with PD in the current study, it does not appear to be a driving factor of the adaptive responses in this sample. However, given that individuals with PD demonstrate difficulty in correcting for sensorimotor errors during self-generated movements [76–78], we cannot rule out the possibility that the perception during self-productions (autophonic judgments) may have contributed to the atypical adaptive responses in PD. Previous work has found that when listening to their own voice playback, individuals with PD are able to determine the vocal loudness within normal limits [79], whereas during an active speech task, individuals with PD overestimated their own vocal loudness [80]. Additionally, Huang, Chen [47] revealed differences at the cortical level between individuals with PD and healthy controls during an active speech task with frequency-altered-feedback, but no differences were seen when individuals with PD were passively listening to the playback of their own voice.

The atypical responses seen in the current study could also be due to a deficit in the feedback system in correcting the detected errors. However, because studies of reflexive responses to sudden, brief, perturbation of *f*_o_ have demonstrated that speakers with PD have *larger* compensatory response magnitudes [30, 32, 33] relative to control speakers, there is evidence that the ability to produce corrective responses to detected errors during auditory perturbations is not impaired in PD. Thus, it is unlikely that the current results are due to an inability of the feedback system to compensate for detected errors in *f*_o_. The larger reflexive compensations in the PD group seen by Liu, Wang [33] were in response to a 100 cent *f*_o_ perturbation and Chen, Zhu [32] reported similar results with 50, 100 and 200 cent perturbation conditions. Additionally, Chen, Zhu [32] found an increase in response magnitude as perturbation size increased. Mollaei, Shiller [30] also observed that a PD group had larger compensatory response gain as compared to controls when the perturbation was 50 cents, but found no group difference in compensatory response gain when the perturbation was 100 cents. Larger compensation magnitudes suggest that the feedback system is not impaired and that, in fact, there may be a higher reliance on the feedback system in PD than in healthy speakers during reflexive responses to *f*_o_. It is possible that speakers with PD rely more on their auditory feedback for *f*_o_ control because of a feedforward system impairment. Additionally, these larger compensations may be due to a deficiency in the weighting of somatosensory and auditory feedback in PD. This weighting in known to vary in typical speakers, but more extreme variation could explain the results shown here [28].

The current results, together with previous work, most directly support the possibility that *f*_o_ control in speakers with PD is impaired due to a deficit in the updating of the feedforward control system after an error signal has been correctly generated. This would mean the *f*_o_ changes are accurately detected and corrected by the feedback control system in speakers with PD, but these corrective responses are not properly subsumed into the feedforward system. This interpretation aligns with previous work suggesting the basal ganglia have a role in generating feedforward commands [81–83] and that the striatum (the primary target of cortical projections to the basal ganglia) contributes to motor learning processes [84]. Therefore, the reduced dopaminergic signals in the striatum seen in PD [85, 86] would impact the integration of corrective motor commands into consequent motor actions. This is further supported by studies which found atypical sensorimotor integration [63, 87–91], including auditory-motor integration [31], in PD. To clarify the contributions of the feedback and feedforward system to vocal motor control in speakers with PD, a comprehensive experiment with both unexpected and sustained perturbations of *f*_o_ is necessary.

### Limitations and future work

A limitation of the current study, as in most studies in PD, is the heterogeneity in the presentation of the speakers with PD in terms of the timing of the onset of their symptoms and the severity of their symptoms. Individuals with PD reported with a wide range in the time between the onset of PD symptoms and their participation in this study (1.5–30 years), as well as in their UPDRS total motor score (ranging from 22–72). The variability in disease severity is difficult to circumvent in studies of PD, as there are a wide range of motor and non-motor symptoms seen in the clinical population of individuals with PD [92–95]. However, progression of dysprosody in PD does not appear to correlate with disease duration or severity as assessed by the UPDRS motor score [96, 97].

Medication state may also have influenced the current study. All speakers with PD were tested while they were receiving L-dopa therapy which allows for variability in duration of administration as well as the individual’s symptoms at the time L-dopa therapy was initiated, influencing the effects of the medication [98–102]. Previous research on speech production in PD has not demonstrated substantial differences in mean *f*_o_ [103–106] or *f*_o_ variability [97, 105, 107] between participants on and off medication. However, during brief perturbations of *f*_o_, larger responses were reported in speakers with PD off medication as compared to a healthy control group [30, 32, 33], whereas one study [34] found that ten speakers with PD on medication did not differ from ten control speakers in terms of reflexive response magnitude. Speakers with PD were not directly compared on and off medication in these studies, but the combined results imply there could be an effect of medication on auditory-motor control. In summary, the effects of medication state on adaptive responses in the auditory domain are not defined, especially given the heterogeneity of responses to medication in the PD population, and should be further investigated by examining adaptive responses of participants who are both on and off medication. The perception of reduced fluctuation in voice fundamental frequency, or ‘monopitch’, observed in PD may also have affected the PD group’s ability to change their *f*_o._ However, we would expect such an effect to show reduced or absent responses while the PD group, on an individual level, showed responses with magnitudes comparable to the control group. Another factor that could have contributed to variation in the current study is the cognitive function of individuals with PD. Previous studies have shown that as attentional demands increase, control speakers have reduced responses to both sudden *f*_o_ perturbation [108] and gradual, predictable, *f*_o_ perturbations [109]. Many individuals with PD have cognitive deficits, particularly in attention [110–112]. However, attention deficits in PD appear to mainly affect dual-attention or divided attention tasks [113, 114], and therefore were most likely not a concern for the current study because it was a single task experiment.

Lastly, there was a 45 ms delay in auditory feedback during the experimental procedure. To our knowledge, only one study has examined the impact of delay on adaptive responses to auditory perturbations. Max and Maffett [115] conducted a formant adaptation task under various delay conditions: no delay, 100 ms, 250 ms, and 500 ms delay. Results showed auditory-motor adaptation was eliminated in all delay conditions. Unfortunately, they did not examine delays between 0 and 100 ms. The healthy controls in the current study showed clear adaptive responses and did not exhibit any speech dysfluencies, suggesting auditory-motor adaptation was not affected by the 45 ms experimental delay. However, it is important to note that the impact of feedback delay on speakers with PD compared to control speakers is unclear and requires further investigation.

## Conclusion

In summary, the current study demonstrated that speakers with PD have reduced mean responses to gradually applied, sustained perturbations of *f*_o_, with heterogeneous individual responses. These findings indicate that there may be an impairment in the adaptive control of voice in PD. Furthermore, the current study failed to find a significant difference in pitch acuity between speakers with PD and control speakers. Taken together with previous work, the results suggest that *f*_o_ changes can be accurately detected and corrected by the auditory feedback control system in speakers with PD, but that there is a deficit in the updating of the feedforward control system. A comprehensive study of reflexive and adaptive responses to *f*_o_ and F1 perturbations, both on and off medication, is needed to better characterize the potential deficits in auditory-motor integration associated with PD. These responses, paired with auditory acuity to *f*_o_ and F1, should be investigated with relation to PD symptom severity and functional communication outcomes. These studies would clarify the underlying deficits in feedforward and feedback speech motor control associated with PD and inform the development of targeted treatments for this disorder.

## Supporting information

S1 File Individual speaker data. Individual speaker data by group including sex, age, PD severity, average fundamental frequency, pitch acuity scores, intelligibility scores, compensation magnitudes in cents, baseline variability in cents, and individual trial data for all adaptation conditions.
(XLSX)
